# Ternary superconducting hydrides stabilized via Th and Ce elements at mild pressures

**DOI:** 10.1016/j.fmre.2022.11.010

**Published:** 2022-12-23

**Authors:** Qiwen Jiang, Zihan Zhang, Hao Song, Yanbin Ma, Yuanhui Sun, Maosheng Miao, Tian Cui, Defang Duan

**Affiliations:** a State Key Laboratory of Superhard Materials, College of Physics, Jilin University, Changchun 130012, China; b Institute of High Pressure Physics, School of Physical Science and Technology, Ningbo University, Ningbo 315211, China; c College of Physics, Harbin University of Science and Technology, Harbin 150080, China; d Department of Chemistry and Biochemistry, California State University Northridge, Los Angeles 91330, United States

**Keywords:** High pressure, Hydrides, Superconductivity, *Ab initio* calculations, Electron-phonon coupling

## Abstract

The discovery of covalent H_3_S and clathrate structure LaH_10_ with excellent superconducting critical temperatures at high pressures has facilitated a multitude of research on compressed hydrides. However, their superconducting pressures are too high (generally above 150 GPa), thereby hindering their application. In addition, making room-temperature superconductivity close to ambient pressure in hydrogen-based superconductors is challenging. In this work, we calculated the chemically “pre-compressed” Be-H by heavy metals Th and Ce to stabilize the superconducting phase near ambient pressure. An unprecedented ThBeH_8_ (CeBeH_8_) with a “fluorite-type” structure was predicted to be thermodynamically stable above 69 GPa (76 GPa), yielding a *T*_c_ of 113 K (28 K) decompressed to 7 GPa (13 GPa) by solving the anisotropic Migdal–Eliashberg equations. Be-H vibrations play a vital role in electron–phonon coupling and structural stability of these ternary hydrides. Our results will guide further experiments toward synthesizing ternary hydride superconductors at mild pressures.

## Graphical abstract

**Figure:**
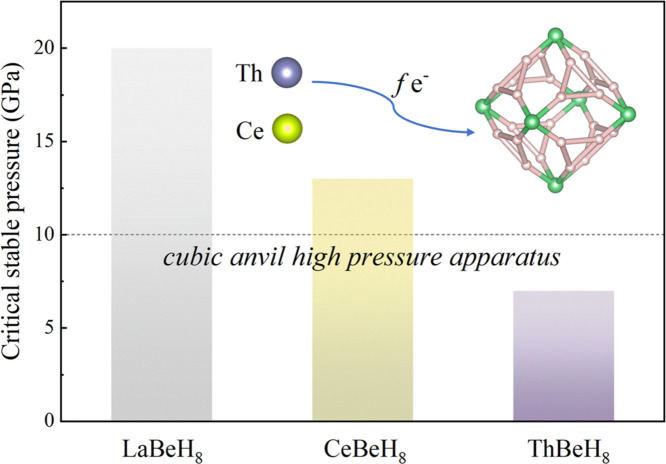

## 1 Introduction

Since the discovery of the superconductivity of mercury in 1911 [1], searching for room-temperature superconductors is an ultimate goal in condensed matter physics and materials science. Within 110 years of exploration, researchers discovered many high critical temperature (*T*_c_) superconductors [2], [3], [4], [5], [6], such as cuprate, iron-based, interface, and organic superconductors. The highest *T*_c_ of 164 K was achieved in Hg-Ba-Ca-Cu-O [7] through diamond anvil cell (DAC) compression to 31 GPa, but the *T*_c_ value remains lower than room temperature.

On the contrary, metallic hydrogen is predicted to be a room-temperature superconductor, but its metallization pressure is high (maybe more than 500 GPa), and it has not been achieved in the laboratory [8,9]. Hydrogen-rich compounds can metallize at lower pressures because of their chemical “pre-compression” effect, which provide an alternative [10]. In 2014, phonon-mediated high-temperature superconductivity was discovered in compressed hydrogen sulfide (203 K for H_3_S at 155 GPa) [11], [12], [13], [14], reigniting the century-old dream of room-temperature superconductivity. Subsequently, sodalite-like LaH_10_ (250 K at 170 GPa) [15], [16], [17], YH_6_ (224 K at 166 GPa) [18,19], YH_9_ (243 K at 201 GPa) [19], and CaH_6_ (215 K at 172 GPa) [20,21] were synthesized. These results promoted the vigorous development of hydrogen-rich superconductors under high pressures. To date, the superconductivity of nearly all binary hydrides has been systematically explored [22], [23], [24], and some hydrides are waiting for experimental confirmation, for example, a layered stacked pentagraphene-like structure HfH_10_ [25] and sodalite-like structure LuH_6_ [26].

Compared with binary hydrides, ternary hydrides have a more complex combination of morphologies, some of which have exhibited favorable superconducting properties. Recently, scientists have synthesized lanthanum–yttrium alloy decahydride with a *T*_c_ of ∼253 K at 183 GPa [27] and theoretically predicted “Hot” superconductivity with a *T*_c_ of 473 K in Li_2_MgH_16_ [28] at a minimum dynamically stable pressure of 250 GPa. The above-mentioned research results show that regardless of binary or ternary hydrides, their superconducting pressure is lower than that of metal hydrogen because of chemical “pre-compression”, but it remains high, usually above 150 GPa.

The experimental conditions for finding high-*T*_c_ hydride superconductors under such pressures are harsh, and only a few groups can participate in this experiment to date. In achieving the pressure above megabar, the DAC with a culet of 40–80 µm was used [13,15]. Sophisticated experimental techniques have caused great difficulties not only to synthesis but also to electrical and magnetic measurements. Thus, balancing the required pressure and predicted *T*_c_ is necessary, rather than solely focusing on maximizing *T*_c_. Another challenge is the pursuit of high-*T*_c_ or room-temperature superconductors in hydrides under moderate pressure or even ambient pressure.

Recently, researchers proposed two possible ways to reduce the stable pressure of hydride superconductors. First, elements with occupied *f*-subshell electrons were considered to be great “pre-compressors”. Based on previous reports, the predicted stable pressures of the same structures are generally lower when *f* electrons were introduced, for example, the predicted stable pressure of YbH_6_ with *f*-electrons is lower than that of CaH_6_ and YH_6_ [26]. Experimentally, ThH_9_ [29], ThH_10_ [29], CeH_9_ [30], [31], [32], CeH_10_ [30], PrH_9_ [33] and NdH_9_ [34] with *f*-electrons have lower stability pressures than LaH_10_. In particular, the pressure of the superhydrides Ce and Th is below 1 megabar. Second, Zhang et al. proposed a strategy that chemically “pre-compressed” covalently bonded hydrogen alloy backbone by heavy atoms could also reduce the superconducting phase pressure [35]. Strikingly, LaBeH_8_ was predicted to be dynamically stable at 20 GPa with a *T*_c_ of 185 K. In addition, LaBH_8_ and KB_2_H_8_ were found to be dynamically stable at ∼50 GPa [36,37] and 12 GPa [38] with *T*_c_ of 126–156 K and 146 K, respectively.

Based on the aforementioned two strategies, we further explored the chemically “pre-compressed” Be-H alloy backbone by selecting the heavy elements Th and Ce with occupied *f*-subshell electrons, which have more pre-compression advantages. Significantly, ThBeH_8_ and CeBeH_8_ with a “fluorite-type” structure are dynamically stabilized at 7 and 13 GPa, respectively. These superconducting pressures are lower than those of ThH_10_ and CeH_10_, and they can be accessible to multi-anvil apparatus. *T*_c_ of ThBeH_8_ is estimated to be 113 K using the anisotropic Migdal–Eliashberg equations, which is higher than the boiling point of liquid nitrogen.

## 2 Computational method

We performed the variable–composition structure search in the Th-Be-H and Ce-Be-H systems at 50 and 100 GPa using an ab initio random structure searching code [39] coupled with the Cambridge Serial Total Energy Package code [40]. In each system, we focused on hydrogen-rich stoichiometries (Th, Ce = 1–2, Be = 1–2, H = 3–12) using primitive cell that consists of 1–2 formula units. For the thermodynamically stable compositions, we re-performed structure search with 1–4 formula units. For ThBH_8_ and CeBH_8_, we modeled cells up to four formula units. A plane wave cutoff energy of 400 eV, a Brillouin zone sampling grid with a spacing of 2π × 0.07 *Å*^−1^, and the Perdew–Burke–Ernzerhof [41] exchange-correlation functional were used in the structure search. The most favorable structures within 50 meV/atom from the hull were relaxed at a higher level of accuracy with cutoff of 800 eV and a k-point grid spacing of 2π × 0.03 *Å*^−1^.

Furthermore, we performed structural relaxations and electronic property calculations of selected phases using the Vienna ab initio simulation package [42] with a kinetic energy cutoff of 800 eV. The projector-augmented wave approach (PAW) [43] was adopted to describe ion–electron interactions, where 1*s*^1^, 2*s*^2^, 2*s*^2^2*p*^1^, 6*s*^2^6*p*^6^5*f*^1^6*d*^1^7*s*^2^, and 5*s*^2^5*p*^6^4*f*^1^5*d*^1^6*s*^2^ are considered as valence electrons for H, Be, B, Th, and Ce atoms, respectively. The crystal orbital Hamiltonian population (COHP) and its integral (ICOHP) were calculated using the LOBSTER code [44]. The zero-point energy (ZPE) was calculated by using the frozen-phonon method with the PHONOPY algorithm [45].

Lattice dynamics and electron–phonon coupling (EPC) calculations were performed using the QUANTUM-ESPRESSO package [46]. A kinetic energy cutoff of 80 Ry and ultrasoft pseudopotentials were adopted in Th-Be-H and Th-B-H systems, and 100 Ry and PAW potentials were adopted in Ce-Be-H and Ce-B-H systems. In addition, we tested the validity of ultrasoft pseudopotentials and PAW potentials (Fig. S1). In the isotropic EPC calculation, the number of k-points and q-points was 24 × 24 × 24 and 6 × 6 × 6 for *Fm*$\bar{3}$*m* phases, 20 × 20 × 16 and 5 × 5 × 4 for *P*6_3_/*mmc*-ThBeH_6_, and 24 × 24 × 24 and 6 × 6 × 6 for *R*$\bar{3}$*m*-ThBeH_6_. The anisotropic electron–phonon Eliashberg functions for ThBeH_8_ and CeBeH_8_ were calculated using Wannier-based interpolation on a 60 × 60 × 60 k-point mesh and a 30 × 30 × 30 q-point mesh, as implemented in the Electron–phonon Wannier code [47].

## 3 Results and discussion

**Fig. 1:**
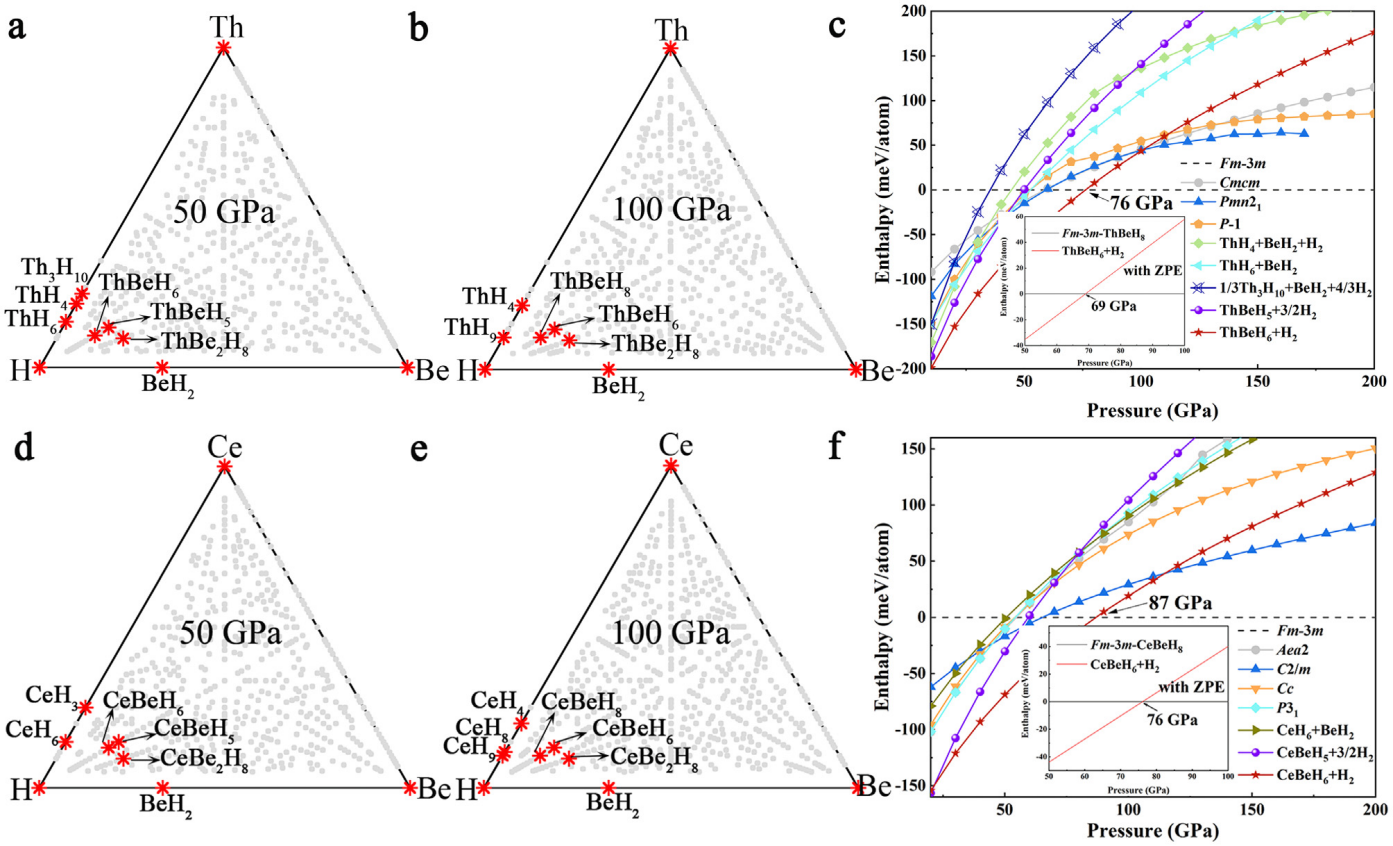
**The ternary phase diagram of Th-Be-H system at (a) 50 and (b) 100 GPa and Ce-Be-H system at (d) 50 and (e) 100 GPa.** Red stars indicate stable phases while gray circles indicate metastable phases. Calculated enthalpy as a function of pressure for various structures relative to the (c) *Fm*$\bar{3}$*m*-ThBeH_8_ and (f) *Fm*$\bar{3}$*m*-CeBeH_8_ phase. Insets in (c) and (f): calculated enthalpies with inclusion of ZPE as a function of pressure. All the binary hydrides and some of the elements in the figure are from Refs. [29,31,32,48,49,55].

First, we performed structural predictions at 50 and 100 GPa for ternary Th-Be-H and Ce-Be-H compounds and constructed the high-pressure ternary phase diagram (Fig. 1). Based on the minimum energy principle, all stable ternary hydrides located on the convex hull against decomposition into pure elements or other energetically favorable binary and ternary phases, which are conducive to experimental synthesis and our research focus.

**Fig. 2:**
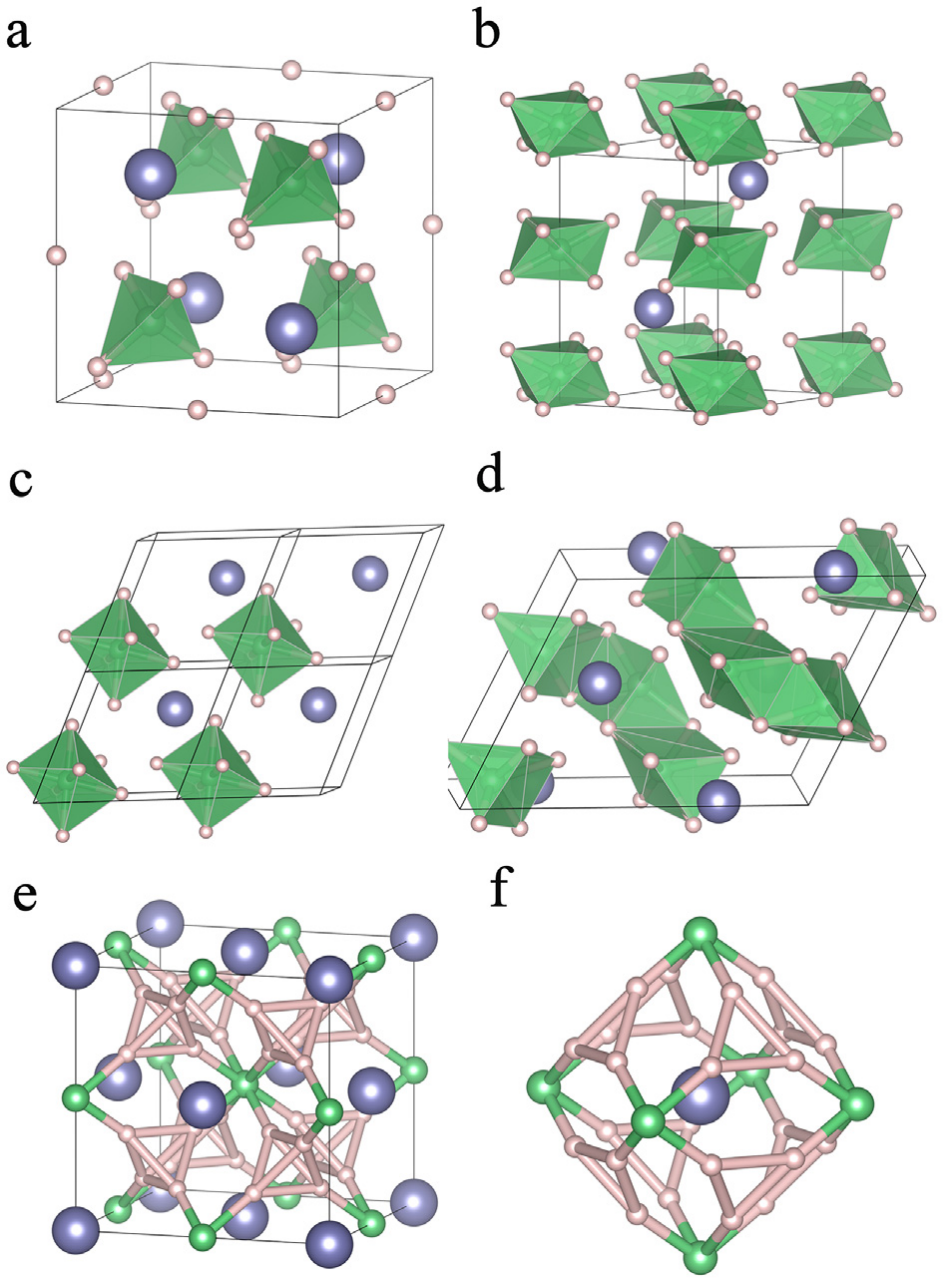
**The crystal structures of (a) *F***$\bar{4}$**3*m*-ThBeH_5_, (b) *P*6_3_/*mmc*-ThBeH_6_, (c) *R***$\bar{3}$***m*-ThBeH_6_, (d) *C*2/*c*-ThBe_2_H_8_ (e) *Fm***$\bar{3}$***m*-ThBeH_8_, and (f) ‘fluorite-type’ cage in ThBeH_8_**.

For the Th-Be-H system at 50 GPa (Fig. 1a), three stable stoichiometries, namely, ThBeH_5_, ThBeH_6_, and ThBe_2_H_8_, were found on the convex hull. For *F*$\bar{4}$3*m*-ThBeH_5_, a single H atom is trapped in the center of the Th tetrahedron, and the remaining H and Be form a BeH_4_ tetrahedron (Fig. 2a). For ThBeH_6_, it has two competitive phases, a ground-state *P*6_3_/*mmc* phase and a metastable *R*$\bar{3}$*m* phase, whose enthalpy value is only 4 meV/atom higher than that of the *P*6_3_/*mmc* phase. In the *P*6_3_/*mmc* structure, six H atoms are incorporated in the Be-H sublattice to form parallel BeH_6_ octahedron, layered with Th (Fig. 2b). In addition, the atomic arrangement of the *R*$\bar{3}$*m* phase is similar to that of *P*6_3_/*mmc* (Fig. 2c), which can be regarded as the distorted *P*6_3_/*mmc*. In BeH_2_ [48], this only stable beryllium hydride is ranged in layers of edge-sharing BeH_6_-octahedra. By contrast, ThBe_2_H_8_ adopts a monoclinic structure with *C*2/*c* symmetry, and it is composed of edge-sharing BeH_6_-irregular pentahedron (Fig. 2d).

At 100 GPa (Fig. 1b), a new hydride (ThBeH_8_) appears on the phase diagram, whereas ThBeH_5_ becomes metastable, and ThBeH_6_ and ThBe_2_H_8_ remain in the observed motifs. In particular, ThBeH_8_ adopts *Fm*$\bar{3}$*m* symmetry, an isostructure with LaBeH_8_, where H_4_ tetrahedral unit fills the tetrahedral voids of the cubic lattice, and Be occupies the center of the octahedral voids (Fig. 2e, 2f). The thermodynamically stable pressure of ThBeH_8_ can be determined by calculating the enthalpy curve (Fig. 1c). *Fm*$\bar{3}$*m*-ThBeH_8_ is the most stable phase against decomposition into ThBeH_6_ and H_2_ above 76 GPa. Considering ZPE, the thermodynamically stable pressure drops to 69 GPa. Further calculation of the phonon dispersion curve found no imaginary frequency down to 7 GPa, exhibiting dynamic stability under quite low pressure (Fig. S2).

As might be anticipated, the Ce-Be-H ternary phase diagrams have a great resemblance with Th-Be-H, and identical stoichiometries appear on the convex hull diagram at 50 and 100 GPa (Fig. 1d, 1e). The predicted stable crystal structures of the Ce-Be-H system are shown in Fig. S3. We also obtain CeBeH_8_ with *Fm*$\bar{3}$*m* symmetry, which can be thermodynamically stable above 87 GPa and down to 76 GPa including ZPE, with a minimum dynamically stable pressure of 13 GPa (Figs. 1f and S2). Such a low pressure (∼10 GPa) can be achieved using the multi-anvil apparatus with centimeter-sized sample volume, reducing the difficulty of synthesis and measurement of physical properties. Thus, ThBeH_8_ and CeBeH_8_ are potential candidates for the exploration of superconductivity under low pressures.

**Fig. 3:**
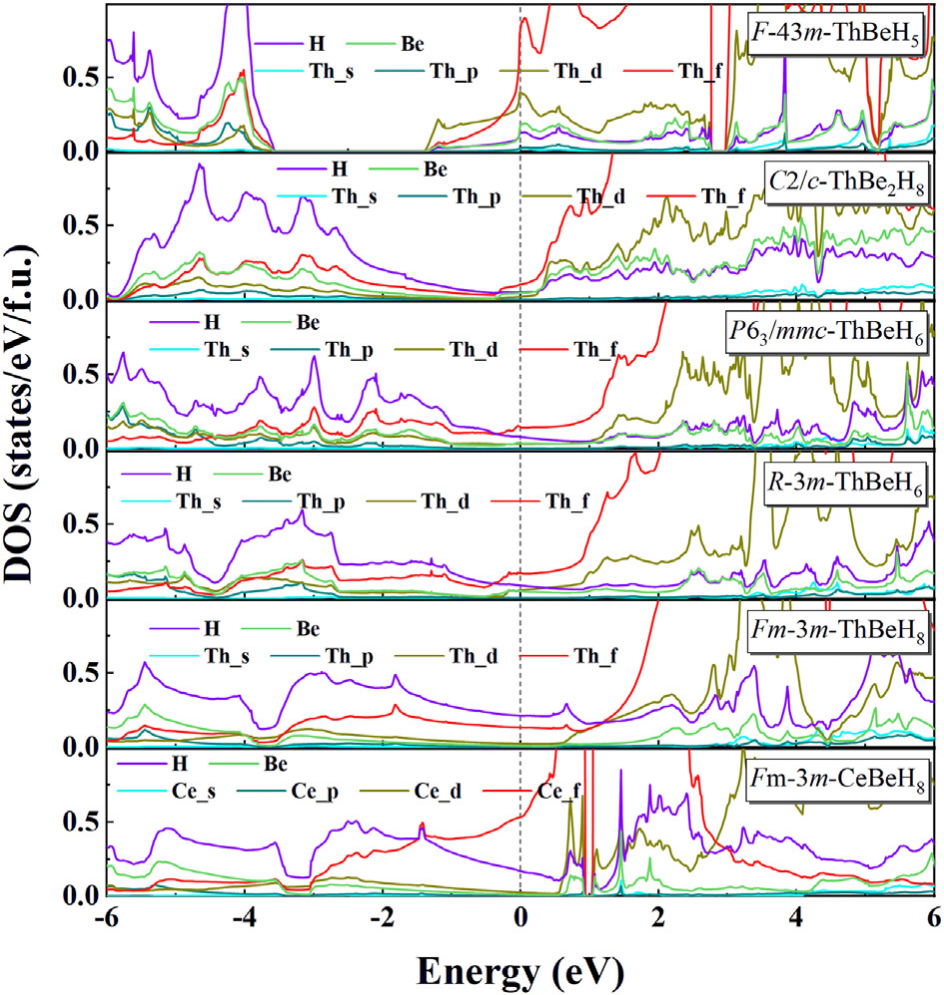
**Electronic density of states of *F***$\bar{4}$**3*m*-ThBeH_5_, *C*2/*c*-ThBe_2_H_8_, *P*6_3_/*mmc*-ThBeH_6_, *R***$\bar{3}$***m*-ThBeH_6_, *Fm***$\bar{3}$***m*-ThBeH_8_ and *Fm***$\bar{3}$***m*-CeBeH_8_ at 50 GPa**.

We calculated the electronic band structures and electronic density of states (DOS) of the stable Th-Be-H and Ce-Be-H compounds at 50 GPa (Figs. 3 and S4, S5). All the proposed phases are metallic, and the characteristics of the electronic structure at the Fermi surface will affect their superconductivity behavior. Based on previous reports, high *T*_c_ hydride superconductors have some common properties [25], that is, high symmetrical structure, electronic DOS at the Fermi level dominated by H atoms, and strong EPC. ThBeH_5_ has a highly symmetrical cubic structure, but a high DOS value at the Fermi level is provided by *d* and *f* states of Th. For the low symmetrical monoclinic *C*2/*c*-ThBe_2_H_8_, the electronic DOS at the Fermi level is primarily derived from *f* state of Th. Similarly, this significant *f*-state occurs in CeBeH_5_, CeBeH_6_, and CeBe_2_H_8_, accompanied by weak H-state occupation, which are closely related to the partial filling of 4*f* electrons in Ce atoms (Fig. S5). The high *f*-state dominance has an adverse effect on superconductivity [26,34]. Therefore, the superconductivity of ThBeH_5_, ThBe_2_H_8_, CeBeH_5_, CeBeH_6_, and CeBe_2_H_8_ showed no importance. In the two phases of ThBeH_6_, as *f* states are pulled away from the Fermi level, the proportion of H DOS at the Fermi level increases to ∼25%. By contrast, in ThBeH_8_, the DOS value of H atoms at the Fermi level constitutes the main contribution (55.3%). For CeBeH_8_, the DOS value of H atoms at the Fermi level is nearly similar to that of ThBeH_8_, but localized *f* states near the Fermi level could suppress its superconductivity (Fig. S6).

**Fig. 4:**
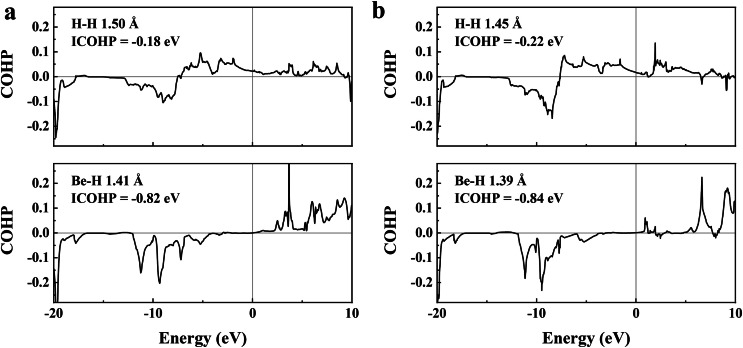
**The calculated COHP and ICOHP of Be-H and H—H bonds of (a) ThBeH_8_ and (b) CeBeH_8_ at 100 GPa**. The negative ICOHP values represent the bonding interactions and positive ICOHP values represent the anti-bonding interactions.

Here, we focus on the cubic phase of ThBeH_8_ and CeBeH_8_. In particular, ThBeH_8_ has a greater H atom contribution to the Fermi level. We examine the bonding feature of ThBeH_8_ and CeBeH_8_ using Bader charge analysis (Table S1), COHP and ICOHP (Fig. 4), and electron localization functions (ELF, Fig. S7). Bader analysis of ThBeH_8_ at 100 GPa reveals that Th and Be atoms transfer a large charge of 1.81 |e| and 1.59 |e| to H atoms; thus, each H atom adopts 0.43 |e|. For CeBeH_8,_ the donated charge of Be (1.57 |e|) is similar to that of ThBeH_8_, but Ce atoms donate 1.39 |e|, thereby causing each H atom to accept 0.37 |e|. The additional charge transferred to H atoms occupies the anti-bonding σ* orbital to weaken the H—H bonds and increase the contribution of H DOS at the Fermi level. At 100 GPa, the shortest H—H distances in both phases (1.50 Å in ThBeH_8_ and 1.45 Å in CeBeH_8_) are larger than that of H_2_ molecules (0.74 Å) [49], and the negative ICOHP values (approximately −0.18 to −0.22 eV) show weak bonding interaction combined with their ELF analysis. On the other hand, the Be-H distances in ThBeH_8_ and CeBeH_8_ at 100 GPa are 1.41 Å and 1.39 Å, respectively, which are almost the same as the covalent bond length (1.42 Å) of the low-pressure phase of *P*$\bar{3}$*m*1-BeH_2_. In addition, the ICOHP value of the Be-H bonds is approximately −0.8 eV, which is four times as large as that of the H—H bonds (−0.2 eV), indicating that Be-H bonds have stronger bonding interactions. All the Be-H states below the Fermi level are bonding, whereas H—H has partial anti-bonding states. The bonding states occupied by Be-H bonds with low energy are more stable than the antibonding states occupied by H—H bonds. Thus, compared with binary hydride-rich superconductors ThH_10_ and CeH_10_, the ternary hydrides ThBeH_8_ and CeBeH_8_ via the introduction of the Be-H bonds contribute to structural stability at mild pressures.

**Fig. 5:**
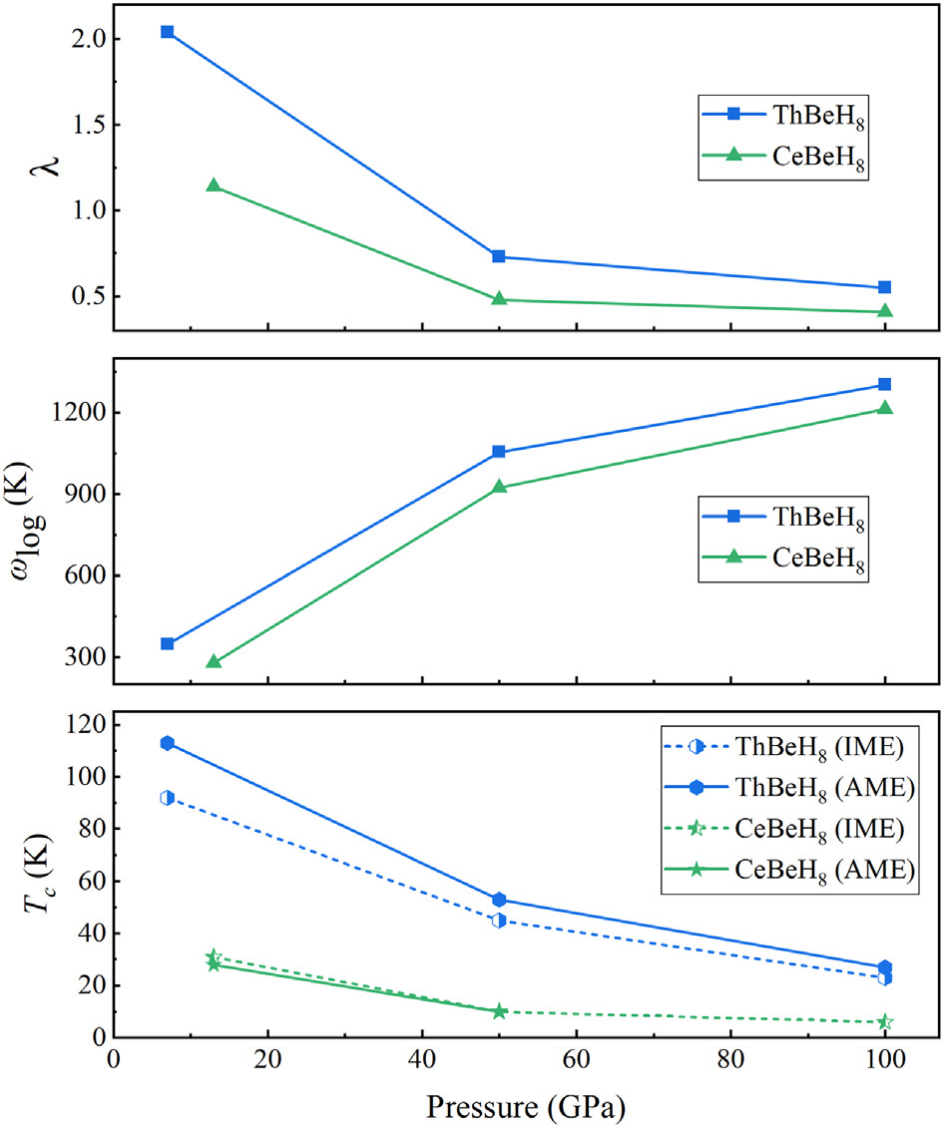
**The calculated EPC parameter λ, logarithmic average phonon frequency *ω*_log_, and *T*_c_ as functions of pressure for ThBeH_8_ and CeBeH_8_**. IME and AME correspond to solutions of the isotropic and anisotropic Migdal-Eliashberg equations, respectively.

To examine the superconductivity of the above-mentioned stable structures, we calculate their *T*_c_s by solving the isotropic Migdal–Eliashberg equations (IME) with typical Coulomb pseudopotential parameters μ* = 0.1–0.13 (Tables S3, S4). The two phases *P*6_3_/*mmc* and *R*$\bar{3}$*m* of ThBeH_6_ exhibit identical *T*_c_ of 48 K (μ* = 0.1) at dynamically stable pressure of 60 and 70 GPa, respectively. The calculated *T*_c_ of ThBeH_8_ is 16–23 K at 100 GPa. With decreasing pressure (Fig. 5), *T*_c_ increases with stronger EPC strength (from 0.55 to 2.04) and finally reaches a maximum value of 92 K using μ* = 0.1 at 7 GPa. For CeBeH_8_, the calculated *T*_c_ is 6 K at 100 GPa and increases to 31 K at 13 GPa. More accurate *T*_c_ values may be obtained by solving the anisotropic Migdal–Eliashberg equations (AME), and *T*_c_ is estimated to be 113 K for ThBeH_8_ at 7 GPa and 28 K for CeBeH_8_ at 13 GPa. Obviously, *T*_c_ values obtained from the AME are close to the IME one (Fig. 5). Thus, the anisotropy of the superconducting gap for ThBeH_8_ and CeBeH_8_ is limited.

Notably, the minimum dynamic stable pressure of 7 GPa in ThBeH_8_ with *T*_c_ over liquid nitrogen temperature is redefined compared with LaBeH_8_ at 20 GPa [35], which is lower than the experimental stabilization pressure baseline of ThH_10_ (85 GPa) [29]. Meanwhile, doping Be into the binary ThH_10_ system reduces the electronic DOS values (almost half) at the Fermi level, thereby leading to a smaller EPC and lower *T*_c_, that is, 161 K for ThH_10_ [29] and 113 K for ThBeH_8_. It is noted that the *T*_c_ of CeBeH_8_ is lower than that of ThBeH_8_. In contrast to the highly delocalized nature of the 5*f* electron in ThBeH_8_, the narrow bands of 4*f* electrons in CeBeH_8_ reinforces its inhibitory effect on superconductivity. In achieving the effect of *f* electrons in Ce atoms on superconductivity, we calculated *T*_c_ of CeBeH_8_ using the Ce pseudopotential with *f*-electron as core in an extreme case, yielding a high *T*_c_ of 198 K, a large *λ* of 3.31, and a high dynamical pressure of 30 GPa (Fig. S8; Table S2). Introducing *f*-electrons can lead to a great reduction in EPC, thereby decreasing *T*_c_ and reducing stable pressure.

Considering the occupancy of electrons in the *f* orbital at the Fermi level, we determined the magnetic properties of the systems at pressure ranging from 0 to 200 GPa (Fig. S9). The results show that all stable Th-Be-H compounds, CeBeH_6_ and CeBeH_8_, are non-magnetic. CeBeH_5_ has strong magnetism moment of 1.0 µ_B_ at ambient pressure, and the magnetism moment decreases with increasing pressure and finally disappears above 90 GPa. *C*2/*c*-CeBe_2_H_8_ only exhibits weak magnetism below 20 GPa.

**Fig. 6:**
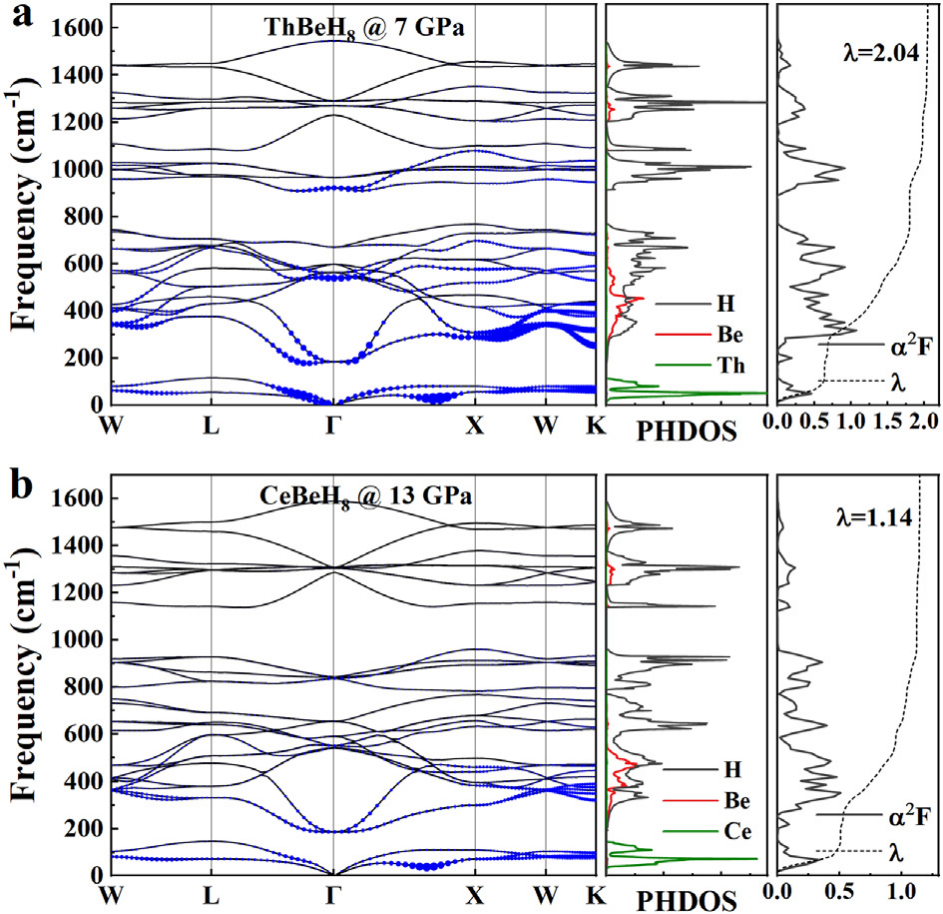
**The calculated phonon dispersion curves, projected phonon densities of states (PHDOS), Eliashberg spectral function and its integral *λ* of (a) ThBeH_8_ at 7 GPa and (b) CeBeH_8_ at 13 GPa**. The radius of the blue solid circle reflects the contribution to EPC.

The phonon spectra, projected phonon DOS, Eliashberg spectral function, and its integral λ of ThBeH_8_ and CeBeH_8_ are calculated, and the results are shown in Fig. 6. ThBeH_8_ exhibits a great EPC strength of 2.04 at 7 GPa, and CeBeH_8_ has a moderate EPC strength of 1.14 at 13 GPa. The phonon mode is primarily divided into three spectrum regions to exhibit different characteristics of EPC. At low frequency (below ∼200 cm^−1^), the heavy Th and Ce atoms contribute 31.4% and 44.3% of the total λ , respectively. The peak in the Eliashberg spectral function at approximately 100 cm^−1^ corresponds to the soft phonon modes along the Γ−X direction, enhancing the EPC strength. Above ∼550 cm^−1^, high-frequency H vibrations contribute only 22.6% and 18.0% to their EPC, respectively, particularly above ∼1,200 cm^−1^, which is quite small. At the middle frequency range of 200–550 cm^−1^, stretching and wagging vibrations of Be-H play a vital role in EPC accounting for 46.0% (ThBeH_8_) and 37.7% (CeBeH_8_). This result is different from Li-P-H [50,51], Li-B-H [52], and K-B-H [38] compounds where pure H atoms dominate the total λ. The stretching and wagging vibration mode of Be and H is conducive to the stability of this structure.

In general, heavier atoms might suppress the superconductivity because of low Debye temperature. On the other hand, heavier atoms are usually accompanied by lower phonon frequencies (soft phonons), which may enhance the strength of EPC [53]. We also performed structure search for ThBH_8_ and CeBH_8_ and sketched their ternary convex hull diagrams at 300 GPa (Fig. S10). The results indicate that ThBH_8_ and CeBH_8_ with *Fm*$\bar{3}$*m* symmetry are ∼10 and ∼16 meV/atom above the convex hull, respectively. For CeBH_8_, the *P*6_3_/*mmc* phase is the most stable structure, and its enthalpy is favorable than *Fm*$\bar{3}$*m* phase until 500 GPa. We calculated the logarithmic average phonon frequency, EPC, and *T*_c_s of *Fm*$\bar{3}$*m*-ThBH_8_ and *Fm*$\bar{3}$*m*-CeBH_8_ under high pressures (Table S3). The result showed that the EPC parameter λ of ThBH_8_ can reach 2.40, which is larger than that of ThBeH_8_. The λ of CeBH_8_ can also reach 1.67 greater than that of CeBeH_8_. The resulting *T*_c_ values are 155 K for ThBH_8_ at 200 GPa and 100 K for CeBH_8_ at 110 GPa using isotropic Migdal–Eliashberg equations. The findings indicate that *T*_c_s values are significantly enhanced. However, the presence of B-H polyhedrons has an adverse effect on the stability, that is, they are thermodynamically unstable up to 300 GPa (> 10 meV above the convex hull). Moreover, the calculation of phonon spectra (Fig. S11) indicates that the minimum dynamically stable pressure of ThBH_8_ and CeBH_8_ is 200 and 110 GPa, respectively, which is higher than that of ThBeH_8_ and CeBeH_8_. These results show that the B-H sublattice can effectively improve the superconducting transition temperature, but it also increases the superconducting stability pressure.

**Fig. 7:**
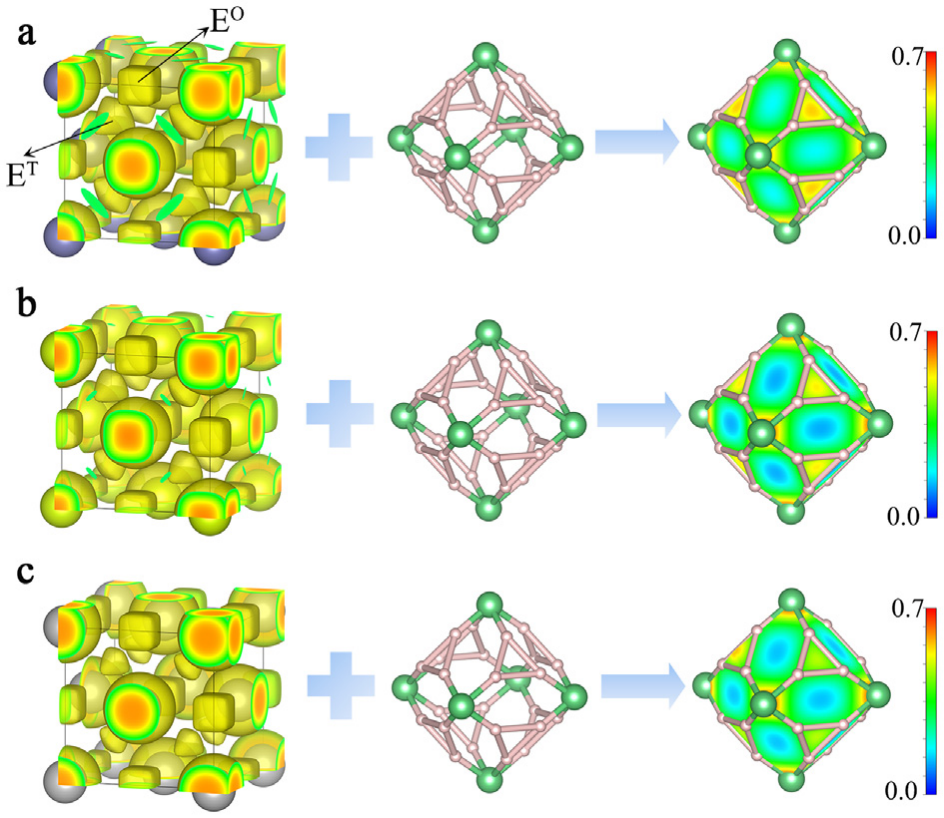
**The ELF (isosurface = 0.4) of (a) the Th lattice in ThBeH_8_, (b) the Ce lattice in CeBeH_8_, and (c) the La lattice in LaBeH_8_ overlaid on the fluorite-type cage at 100 GPa, respectively**.

Similar to many other high-pressure superhydrides such as CaH_6_, YH_9_, and LaH_10_, the stability of XBeH_8_ (*X* = Th, Ce, and La) compounds is also determined by the chemical template effect [54]. As shown by the ELF in Fig. 7, high electron localizations are found at the octahedral E^O^ sites and tetrahedral E^T^ sites of the X metal sublattices, indicating the occupation of the interstitial local orbitals centered at these sites (quasi-atoms). The good match of the quasi-atom orbitals and the local orbitals of the BeH_8_ units provides a strong driving force stabilizing the metal and BeH_8_ sublattices. It is interesting to notice that the ELF values of 0.63 and 0.62 at the E^T^ sites in the Th and Ce lattices, respectively, are considerably higher than that of 0.56 in the La lattice, indicating a stronger chemical template effect. Indeed, ThBeH_8_ and CeBeH_8_ are found to remain stable under lower pressures than LaBeH_8_.

The chemical template effect allows the electrons occupying the quasi-atom orbitals in metal lattices to naturally dope the BeH_8_ lattice, which minimizes the energies of the metal and BeH_8_ lattices. Compared with conventional interactions such as ionic and covalent bonds, the chemical template is relatively weaker. In revealing this mechanism, we calculate two charge density differences, defined as Δ*ρ*_1_ = *ρ*_total_ − *ρ*_X_ − *ρ*_BeH8_ and Δ*ρ*_2_ = *ρ*_total_ − *ρ*_XBe_ − *ρ*_H8_. As displayed in Fig. S12, the charge density differences show only a slight electron transfer among the sublattices, although H is highly electronegative compared with metals. Similar to other superhydrides, H loses its capability of drawing electrons while forming covalent BeH_8_ lattices, and the weak template effect becomes a determining factor of the formation of these superhydrides. Another important feature of the chemical template effect is that it strongly depends on metals. In contrast to early transition metals that show a strong chemical template effect, late transition metals, such as W, Re, Os, Ir, Pt, and Au (Fig. S13), show a weaker template effect because their covalent electrons bind stronger with the core and show little occupation of the quasi-atom orbitals. Consequently, these metals do not form similar XBeH_8_ compounds.

## 4 Conclusion

We have designed two new hydride superconductors with “pre-compressed” binary Be-H alloy backbone using the heavy metal elements Th and Ce at mild pressures. ThBeH_8_ is thermodynamically stable above 69 GPa and dynamically stable down to 7 GPa, with high *T*_c_ of 113 K under the fully anisotropic ab initio Migdal–Eliashberg theory. To the best of our knowledge, *T*_c_ rarely exceeds the temperature range of liquid nitrogen at such a low pressure in superhydrides. Similar pressure conditions of 13 GPa are also achieved in CeBeH_8_ compound with *T*_c_ of 28 K. The replacement of Be by heavy element B further improves the *T*_c_ values; ThBH_8_ can reach 155 K, and CeBH_8_ can reach 100 K, although the stable pressure is also increased. Furthermore, we use the chemical template effect to comprehensively understand the stability of XBeH_8_ (*X* = Th, Ce and La) at mild pressures. In this work, the ternary superhydrides discovered at low pressure will stimulate multi-anvil presses experimental studies of hydrogen-based superconductors.

## Declaration of competing interest

The authors declare that they have no conflicts of interest in this work.

## Appendix Supplementary materials

Image, application 1
