# Supplementary material for: Ternary superconducting hydrides stabilized via Th and Ce elements at mild pressures
Source: Fundam Res. 2022 Dec 23;4(3):550–6. doi: 10.1016/j.fmre.2022.11.010 (PMC11197597; doi:10.1016/j.fmre.2022.11.010)
Supplement: Supplementary file 1 [file mmc1.docx]

Supplementary Materials

for

Ternary superconducting hydrides stabilized via Th and Ce elements at mild pressures

Qiwen Jiang^a^, Zihan Zhang^a^, Hao Song^b^ Yanbin Ma^c^, Yuanhui Sun^d^, Maosheng Miao^d^, Tian Cui^b,a^, Defang Duan^a,^*

*^a^ State Key Laboratory of Superhard Materials, College of Physics, Jilin University, Changchun 130012, China*

*^b^ Institute of High Pressure Physics, School of Physical Science and Technology, Ningbo University, Ningbo 315211, China*

*^c^ College of Physics, Harbin University of Science and Technology, Harbin 150080, China*

*^d^**Department of Chemistry and Biochemistry, California State University Northridge, Los Angeles,*

*California 91330, United States*

** Corresponding author:* [*duandf@jlu.edu.cn*](mailto:duandf@jlu.edu.cn) (Defang Duan)*.*

**Validity test of pseudopotential**

In all calculations, the choice of pseudopotential is crucial. In order to test the validity of different pseudopotentials, we calculate the equation of states (EOS) of the ThBeH_8_ and CeBeH_8_ using projector-augmented wave (PAW) potentials and on-the-fly generation of ultrasoft pseudopotentials (OTF), and ultrasoft pseudopotentials compared with the all-electron full-potential linearized augmented plane wave (FP-LAPW) method as shown in Fig. S1. For ThBeH_8_,it clearly shows that the EOS calculated from PAW, OTF and ultrosoft potential are consistent with the calculation results obtained by the FP-LAPW method as implemented in the ELK program [1]. For CeBeH_8_, the volume calculated by ultrasoft pseudopotentials as performed in the QUANTUM-ESPRESSO program is larger than the results from the FP-LAPW method, while the other results are consistent. Therefore, in the superconductivity calculation, we chose the PAW potential of cerium considering electronic configuration of 5*s*^2^5*p*^6^4*f*^1^5*d*^1^6*s*^2^.


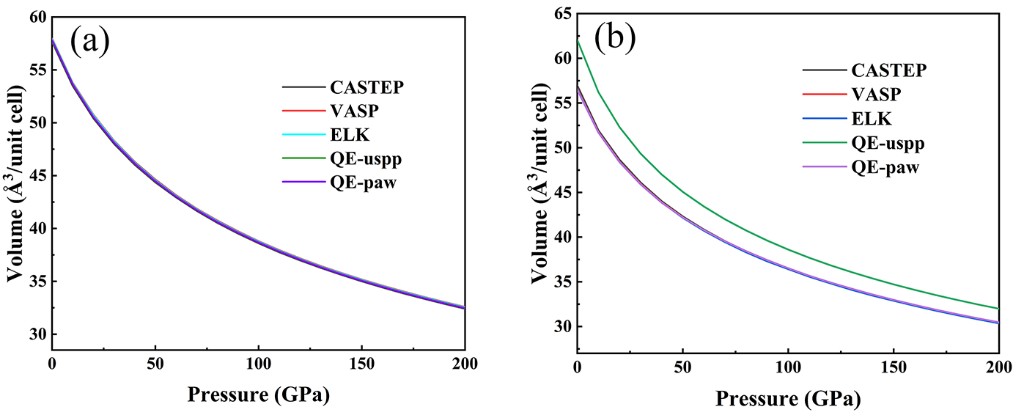


**Fig. S1.** Volumes as a function of pressures for (a) ThBeH_8_ and (b) CeBeH_8_ calculated using the PAW potential in the VASP code, the OTF or ultrasoft pseudopotential in the CASTEP code, the PAW or ultrasoft pseudopotential in the QE code, and the FP-LAPW in the ELK code.

**The crystal structures, electronic properties and phonon dispersion curves of Th-Be-H and Ce-Be-H systems**





**Fig. S2.** The calculated phonon dispersion curves of (a) ThBeH_8_ at 7 GPa and (b) CeBeH_8_ at 13 GPa.


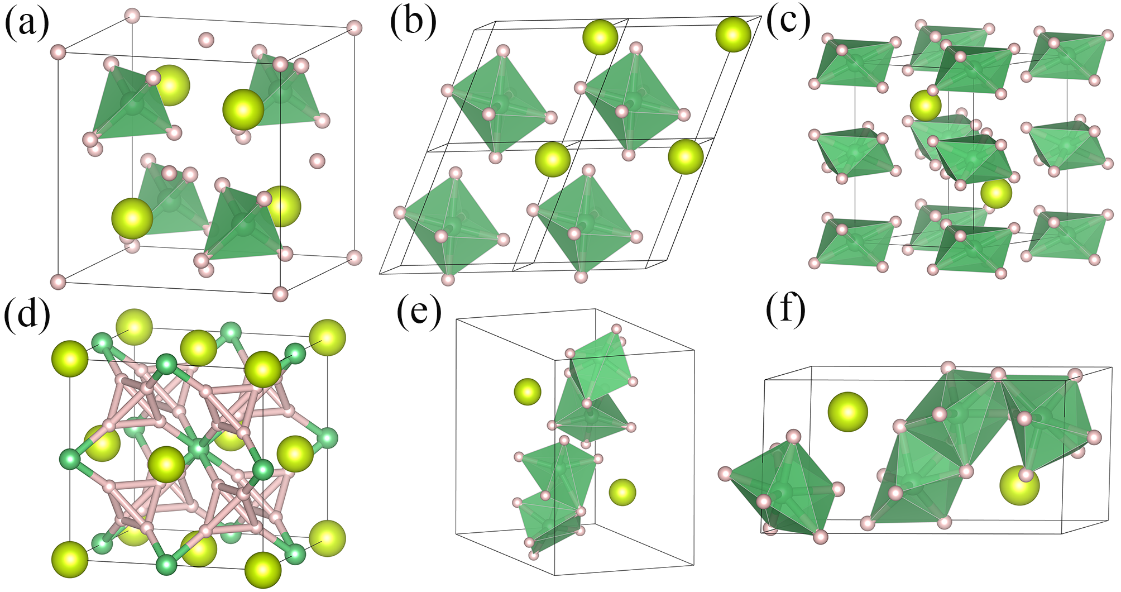


**Fig. S3.** The crystal structures of (a) *F*$\bar{4}$3*m*-CeBeH_5_, (b) *R*$\bar{3}$*m*-CeBeH_6_, (c) *P*6_3_/*mmc*-CeBeH_6_, (d) *Fm*$\bar{3}$*m*-CeBeH_8_, (e) *C*2/*c*-CeBe_2_H_8_ and (f) *Pma*2-CeBe_2_H_8_. The stable Ce-Be-H ternary stoichiometries at 50 GPa are *F*$\bar{4}$3*m*-CeBeH_5_, *R*$\bar{3}$*m*-CeBeH_6_ and *C*2/*c*-CeBe_2_H_8_. The stable Ce-Be-H ternary stoichiometries at 100 GPa are *P*6_3_/*mmc*-CeBeH_6_, *Fm*$\bar{3}$*m*-CeBeH_8_ and *Pma*2-CeBe_2_H_8_.





**Fig. S4.** Calculated electronic band structures and projected density of states (DOS) of (a) *F*$\bar{4}$3*m*-ThBeH_5_, (b) *C*2/*c*-ThBe_2_H_8_, (c) *P*6_3_/*mmc*-ThBeH_6_, (d) *R*$\bar{3}$*m*-ThBeH_6_, (e) *Fm*$\bar{3}$*m*-ThBeH_8_, and (f) *Fm*$\bar{3}$*m*-CeBeH_8_ at 50 GPa.





**Fig. S5.** Calculated electronic band structures and projected DOS of (a) *F*$\bar{4}$3*m*-CeBeH_5_, (b) *R*$\bar{3}$*m*-CeBeH_6_, (c) *P*6_3_/*mmc*-CeBeH_6_, (d) *C*2/*c*-CeBe_2_H_8_, and (e) *Pma*2-CeBe_2_H_8_ at 50 GPa.





**Fig. S6.** Calculated DOS of (a) *Fm*$\bar{3}$*m*-ThBeH_8_ and (b) *Fm*$\bar{3}$*m*-CeBeH_8_. In both figures, the solid line is the data for 50 GPa and the dotted lines are for (a) 7 GPa and (b) 13 GPa, respectively. The total DOS is elevated as the pressure decreases, especially for ThBeH_8_, where this elevation is dependent on the contribution of the H atom. For CeBeH_8_, the *f*-states fraction remains large throughout the studied pressure phase.

Table S1. Bader charge of our predicted stable ternary hydrides at selected pressure.

| Structure | Pressure  (GPa) | Atom | δ (e) |
| --- | --- | --- | --- |
| *R*$\bar{3}$*m*-ThBeH_6_ | 50 | H | -0.59 |
|  |  | Be | +1.55 |
|  |  | Th | +1.98 |
| *P*6_3_/*mmc*-ThBeH_6_ | 100 | H | -0.57 |
|  |  | Be | +1.54 |
|  |  | Th | +1.84 |
| *Fm*$\bar{3}$*m*-ThBeH_8_ | 100 | H | - 0.43 |
|  |  | Be | +1.59 |
|  |  | Th | +1.81 |
| *R*$\bar{3}$*m*-CeBeH_6_ | 50 | H | - 0.51 |
|  |  | Be | +1.54 |
|  |  | Ce | +1.55 |
| *P*6_3_/*mmc*-CeBeH_6_ | 100 | H | - 0.49 |
|  |  | Be | +1.54 |
|  |  | Ce | +1.39 |
| *Fm*$\bar{3}$*m*-CeBeH_8_ | 100 | H | - 0.37 |
|  |  | Be | +1.57 |
|  |  | Ce | +1.39 |
| *Fm*$\bar{3}$*m*-CeBH_8_ | 100 | H | -0.30 |
|  |  | B | +1.04 |
|  |  | Ce | +1.34 |
| *Fm*$\bar{3}$*m*-ThBH_8_ | 100 | H | -0.35 |
|  |  | B | +1.05 |
|  |  | Th | +1.78 |


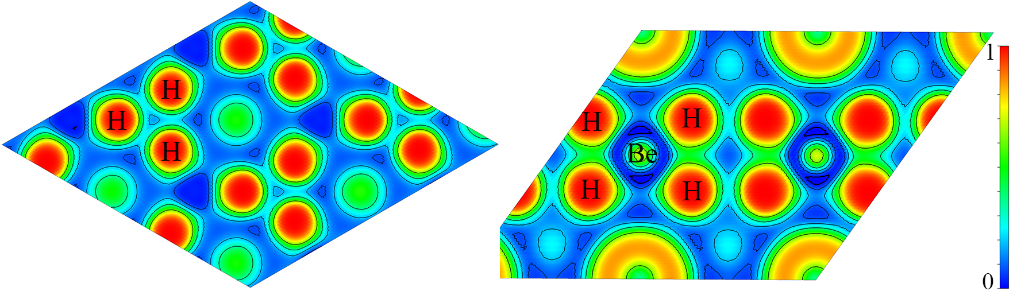


**Fig. S7.** The electron localization functions (ELF) of ThBeH_8_ at 100 GPa along a plane of the H_4_ tetrahedron (left panel) and Be-H plane (right panel).





**Fig. S8.** Phonon dispersion curves, projected phonon densities of states (PHDOS), Eliashberg spectral function and its integral λ of (a) CeBeH_8_ considering *f* electrons as valence electrons at 13 GPa and (b) CeBeH_8_ with frozen-*f* electrons at 30 GPa. The radius of the blue solid circle reflects the contribution to electron- phonon coupling.





**Fig. S9.** Magnetic moments of our predicted stable ternary hydrides under high pressure.

**The convex hull diagrams and phonon dispersion curves of Th-B-H and Ce-B-H systems**


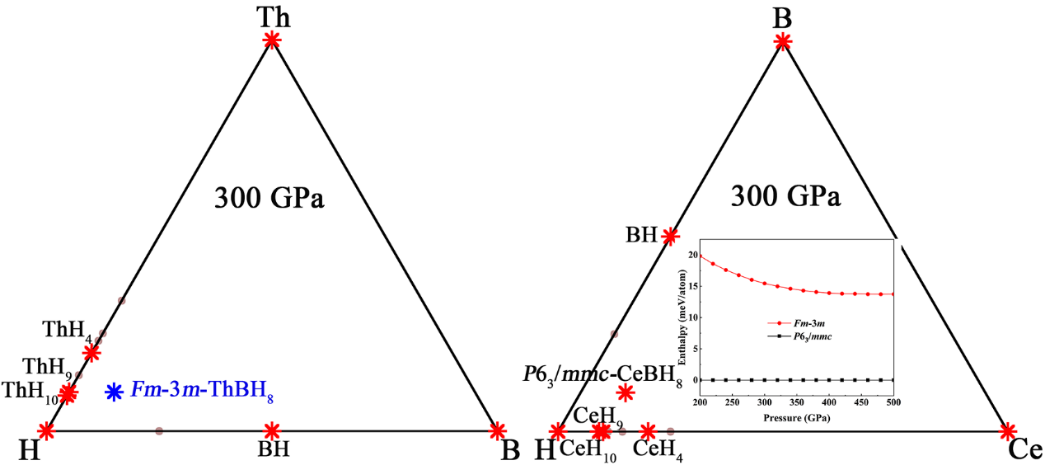


**Fig. S10.** Convex hull diagrams for the Th-B-H system (left) and Ce-B-H system (right) at 300 GPa. Red stars indicate stable phases while grayish brown circles and blue star indicate metastable phases. All the binary hydrides and elements in the figure are from references [2-6]. Our case was simplified by adding the ternary hydrides RBH_8_ (R=Th, Ce) in the phase diagram. The square graph on the right: calculated enthalpy of the *P*63/*mmc* phase relative to the *Fm*$\bar{3}$*m* phase of CeBH_8_ as a function of pressure.





**Fig. S11.** Phonon dispersion curves, projected phonon densities of states (PHDOS), Eliashberg spectral function and its integral λ of (a) ThBH_8_ at 200 GPa and (b) CeBH_8_ at 110 GPa.

**Analysis of the chemical template effect of XBeH_8_ (X = Th, Ce and La)**


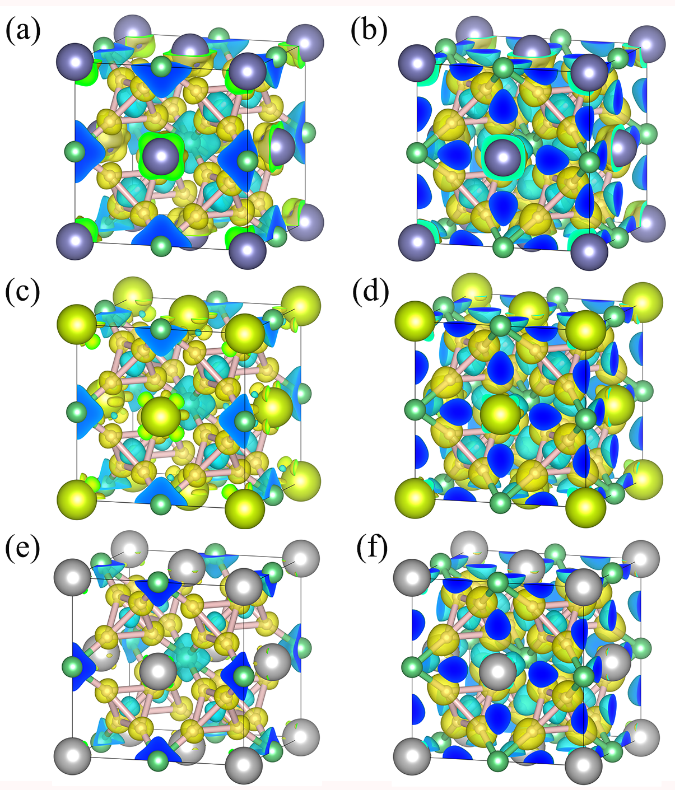


**Fig. S12**. The calculated charge density difference with isosurface value of 0.01 e/ Bohr^3^. (a, c, e) are charge density difference of ThBeH_8_, CeBeH_8_ and LaBeH_8_ respectively, defined as Δ*ρ*_1_ = *ρ*_total_ − *ρ*_X_ – *ρ*_BeH8_ (X = Th, Ce, La). (b, d, f) are charge density difference of ThBeH_8_, CeBeH_8_ and LaBeH_8_ respectively, defined as Δ*ρ*_2_ = *ρ*_total_ − *ρ*_XBe_ − *ρ*_H8_ (X = Th, Ce, La).


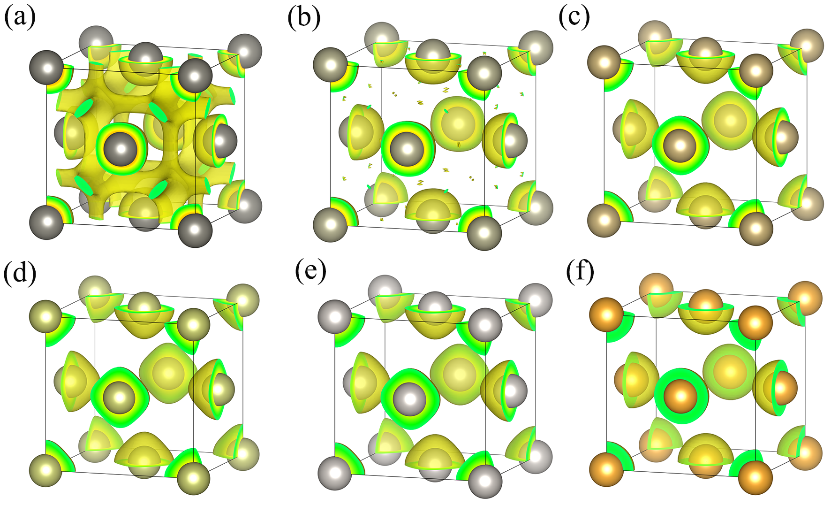


**Fig. S13**. The calculated ELF of (a) WBe_0_H_0_ (b) ReBe_0_H_0_ (c) OsBe_0_H_0_ (d) IrBe_0_H_0_ (e) PtBe_0_H_0_ and (f) AuBe_0_H_0_ with isosurface value of 0.4 at 100 GPa.

**Superconductive parameters and structural information**

Table S2. Calculated superconducting parameters of *Fm*$\bar{3}$*m*-CeBeH_8_ with and without the inclusion of *f* electrons at the corresponding pressure. *T*_c_ was calculated by solving the isotropic Migdal-Eliashberg equations.

| Pressure (GPa) | *λ* | *ω*_log_  (K) | *N_Ef_*  (States/Ry/fu) | *T*_c_ (K) (* μ**=0.1-0.13) |
| --- | --- | --- | --- | --- |
| 13^a^ | 1.14 | 278.6 | 14.52 | 26-31 |
|  |  |  |  |  |
| 30^b^ | 3.31 | 599.1 | 6.61 | 187-198 |
|  |  |  |  |  |

^a^ and ^b^ represent calculations with and without consideration of *f* electrons as valence electrons under their lowest dynamic stability pressure, respectively.

Table S3. The calculated superconducting parameters of our predicted ternary hydrides under high pressures. *T*_c_ was calculated by solving the isotropic Migdal-Eliashberg equations.

| Structure | Pressure (GPa) | *λ* | *ω*_log_  (K) | *N_Ef_*  (States/ /spin/Ry/f.u.) | *T*_c_ (K) (* μ**=0.1-0.13) |
| --- | --- | --- | --- | --- | --- |
| *P*6_3_/*mmc*-ThBeH_6_ | 60 | 1.32 | 428.2 | 6.08 | 43-48 |
|  | 100 | 0.58 | 1056.7 | 6.26 | 16-23 |
| *R*$\bar{3}$*m*-  ThBeH_6_ | 70 | 1.06 | 549.9 | 3.71 | 41-48 |
|  | 100 | 0.66 | 965.1 | 3.67 | 23-31 |
| *Fm*$\bar{3}$*m*-ThBH_8_ | 200 | 2.40 | 759.8 | 7.18 | 146-155 |
|  |  |  |  |  |  |
| *Fm*$\bar{3}$*m*-CeBH_8_ | 110 | 1.67 | 561.9 | 17.17 | 91-100 |
|  |  |  |  |  |  |

Table S4. The calculated superconducting parameters of ThBeH_8_ and CeBeH_8_ under high pressures. *T*_c_^IME^ and *T*_c_^AME^ represent the superconducting transition temperatures obtained by solving the isotropic and anisotropic Migdal-Eliashberg equations respectively.

| Structure | Pressure (GPa) | *λ* | *ω*_log_  (K) | *N_Ef_*  (States/ /spin/Ry/f.u.) | *T*_c_^IME^ (K) (* μ**=0.1-0.13) | *T*_c_^AME^(K) (* μ**=0.1) |
| --- | --- | --- | --- | --- | --- | --- |
| *Fm*$\bar{3}$*m*-ThBeH_8_ | 7 | 2.04 | 347.0 | 4.99 | 84-92 | 113 |
|  | 50 | 0.73 | 1054.1 | 4.10 | 36-45 | 53 |
|  | 100 | 0.55 | 1302.2 | 3.58 | 16-23 | 27 |
| *Fm*$\bar{3}$*m*-CeBeH_8_ | 13 | 1.14 | 278.6 | 14.52 | 26-31 | 28 |
|  | 50 | 0.48 | 923.5 | 8.36 | 5-10 | 10 |
|  | 100 | 0.41 | 1213.8 | 5.70 | 3-6 | / |

Table S5. Structural information of our predicted ternary hydrides.

| **Compound** | **Space group** | **Lattice Parameters**  **(**Å, **°)** | **Atoms** | **Atomic coordinates**  **(fractional)** |
| --- | --- | --- | --- | --- |
| ThBeH_5_  (50 GPa) | *F*$\bar{4}$3*m* | *a* = *b* = *c* = 5.4711  *α* = *β* = *γ =* 90 | H (16e) | 0.60884 -0.60884 -0.89116 |
|  |  |  | H (4b) | 0.50000 -0.50000 -0.50000 |
|  |  |  | Be (4d) | 0.75000 -0.75000 -0.75000 |
|  |  |  | Th (4c) | 0.25000 -0.25000 -0.25000 |
| ThBeH_6_  (50 GPa) | *R*$\bar{3}$*m* | *a* = *b* = 4.0158  *c* = 9.1536  *α* = *β=* 90  *γ* = 120 | H (18h) | -1.48275 -0.51725 0.09525 |
|  |  |  | Be (3b) | -1.33333 0.33333 -0.16667 |
|  |  |  | Th (3a) | -1.66667 -0.33333 -0.33333 |
| ThBeH_6_  (50 GPa) | *P*6_3_/*mmc* | *a* = *b* = 4.0185  *c* = 6.0906  *α* = *β=* 90  *γ* =120 | H (12k) | 0.18447 0.36894 0.10587 |
|  |  |  | Be (2a) | 0.00000 0.00000 0.50000 |
|  |  |  | Th (2d) | 0.33333 0.66667 -0.25000 |
|  |  |  |  |  |
| ThBeH_8_  (100 GPa) | *Fm*$\overline{3}$*m* | *a* = *b* = *c* = 5.3651  *α* = *β* = *γ =* 90 | H (32f) | -0.15119 0.84881 0.34881 |
|  |  |  | Be (4b) | 0.00000 0.50000 0.00000 |
|  |  |  | Th (4a) | 0.00000 0.00000 0.00000 |
| ThBe_2_H_8_  (100 GPa) | *C*2/*c* | *a* = 5.3755  *b* = 5.2999  *c* = 7.1546  *α* = *γ* = 90  *β =* 117.0217 | H (8f) | 1.64135 0.09833 0.98450 |
|  |  |  | H (8f) | 1.84831 -0.47787 1.24785 |
|  |  |  | H (8f) | 2.08236 -0.13789 1.08441 |
|  |  |  | H (8f) | 1.73752 -0.31864 0.86798 |
|  |  |  | Be (8f) | 1.57585 -0.14576 0.91463 |
|  |  |  | Th (4e) | 1.00000 0.15334 0.25000 |
| CeBeH_5_  (50 GPa) | *F*$\bar{4}$3*m* | *a* = *b* = *c* = 5.3714  *α* = *β* = *γ =* 90 | H (16e) | -0.39190 -0.60810 0.60810 |
|  |  |  | H (4a) | -1.00000 -1.00000 1.00000 |
|  |  |  | Be (4c) | -0.25000 -0.25000 0.25000 |
|  |  |  | Ce (4d) | -0.75000 -0.75000 0.75000 |
| CeBeH_6_  (50 GPa) | *R*$\bar{3}$*m* | *a* = *b* = 3.9408  *c* = 8.9029  *α* = *β=* 90  *γ* = 120 | H (18h) | 0.14731 0.29462 -0.75967 |
|  |  |  | Be (3b) | 0.00000 -0.00000 -0.50000 |
|  |  |  | Ce (3a) | 0.00000 -0.00000 -1.00000 |
|  |  |  |  |  |
| CeBeH_6_  (100 GPa) | *P*6_3_/*mmc* | *a* = *b* = 3.7775  *c* = 5.5345  *α* = *β=* 90  *γ* =120 | H (12k) | 0.80836 0.61672 0.10803 |
|  |  |  | Be (2a) | 1.00000 -0.00000 0.00000 |
|  |  |  | Ce (2c) | 0.66667 0.33333 0.75000 |
|  |  |  |  |  |
| CeBeH_8_  (100 GPa) | *Fm*$\overline{3}$*m* | *a* = *b* = *c* = 5.2624  *α* = *β* = *γ =* 90 | H (32f) | -0.84759 -1.15241 0.65241 |
|  |  |  | Be (4b) | -0.50000 -0.50000 0.50000 |
|  |  |  | Ce (4a) | -1.00000 -1.0000 1.00000 |
| CeBe_2_H_8_  (50 GPa) | *C*2/*c* | *a* = 5.5354  *b* = 5.3708  *c* = 7.6240  *α* = *γ* = 90  *β =* 117.4815 | H (8f) | 0.73985 -1.18683 -0.12995 |
|  |  |  | H (8f) | 0.65201 -0.39884 0.48295 |
|  |  |  | H (8f) | 0.58865 -1.13520 -0.41060 |
|  |  |  | H (8f) | 0.34883 -0.47953 -0.24450 |
|  |  |  | Be (8f) | 0.57433 -0.64872 0.41835 |
|  |  |  | Ce (4e) | 0.50000 -0.84138 -0.25000 |
| CeBe_2_H_8_  (100 GPa) | *Pma*2 | *a* = 6.3310  *b* = 3.6665  *c* = 3.6885  *α* = *γ* = *β =* 90 | H (4d) | 0.41736 -0.23454 -0.27505 |
|  |  |  | H (4d) | 0.63213 -0.36622 0.06180 |
|  |  |  | H (2c) | 0.25000 0.72658 -0.61390 |
|  |  |  | H (2a) | 0.50000 0.00000 0.34082 |
|  |  |  | H (2b) | 1.00000 0.50000 0.43668 |
|  |  |  | H (2c) | 0.75000 -0.00864 0.05612 |
|  |  |  | Be (4d) | 0.91584 0.26282 0.09622 |
|  |  |  | Ce (2c) | 0.75000 -0.23956 0.57840 |
| CeBH_8_  (200 GPa) | *Fm*$\overline{3}$*m* | *a* = *b* = *c* = 4.9346  *α* = *β* = *γ =* 90 | H (32f) | -0.84704 0.65296 -0.34704 |
|  |  |  | B (4a) | -0.50000 0.50000 0.00000 |
|  |  |  | Ce (4b) | -0.50000 0.50000 -0.50000 |
| CeBH_8_  (300 GPa) | *P*6_3_/*mmc* | *a* = *b* = 3.3239  *c* = 5.6003  *α* = *β* = 90  *γ =* 120 | H (12k) | 0.19856 0.39711 -0.08175 |
|  |  |  | H (2b) | 0.00000 -0.00000 0.25000 |
|  |  |  | H (2d) | 0.66667 0.33333 0.25000 |
|  |  |  | B (2a) | 1.00000 1.00000 0.50000 |
|  |  |  | Ce (2c) | 0.33333 0.66667 0.25000 |
| ThBH_8_  (100 GPa) | *Fm*$\overline{3}$*m* | *a* = *b* = *c* = 5.2624  *α* = *β* = *γ =* 90 | H (32f) | -0.15241 -0.15241 -0.15241 |
|  |  |  | B (4a) | 0.00000 0.00000 0.00000 |
|  |  |  | Th (4b) | 0.50000 -0.50000 -0.50000 |

**References**

[1] The ELK FP-LAPW code. http://elk.sourceforge.net/

[2] J. M. McMahon, D. M. Ceperley, Ground-state structures of atomic metallic hydrogen. Phys Rev Lett 106. 16. (2011) 165302.

[3] J. McMinis, R. C. Clay, D. Lee, et al., Molecular to atomic phase transition in hydrogen under high pressure. Phys Rev Lett 114. 10. (2015) 105305.

[4] C. J. Pickard, M. Martinez-Canales, R. J. Needs, Density functional theory study of phase IV of solid hydrogen. Physical Review B 85. 21. (2012) 214114.

[5] C. J. Pickard, R. J. Needs, Structure of phase III of solid hydrogen. Nature Physics 3. 7. (2007) 473-476.

[6] C. H. Hu, A. R. Oganov, Q. Zhu, et al., Pressure-induced stabilization and insulator-superconductor transition of BH. Phys Rev Lett 110. 16. (2013) 165504.
